# Exploring the Specific Needs of Persons with Multiple Sclerosis for mHealth Solutions for Physical Activity: Mixed-Methods Study

**DOI:** 10.2196/mhealth.8996

**Published:** 2018-02-09

**Authors:** Guido Giunti, Jan Kool, Octavio Rivera Romero, Enrique Dorronzoro Zubiete

**Affiliations:** ^1^ Salumedia Tecnologias Seville Spain; ^2^ University of Oulu Oulu Finland; ^3^ Kliniken Valens Valens Switzerland; ^4^ Universidad de Sevilla Seville Spain

**Keywords:** multiple sclerosis, telemedicine, fatigue, mobile applications, video games, qualitative research, exercise, chronic disease

## Abstract

**Background:**

Multiple sclerosis (MS) is one of the world’s most common neurologic disorders, with symptoms such as fatigue, cognitive problems, and issues with mobility. Evidence suggests that physical activity (PA) helps people with MS reduce fatigue and improve quality of life. The use of mobile technologies for health has grown in recent years with little involvement from relevant stakeholders. User-centered design (UCD) is a design philosophy with the goal of creating solutions specific to the needs and tasks of the intended users. UCD involves stakeholders early and often in the design process. In a preliminary study, we assessed the landscape of commercially available MS mobile health (mHealth) apps; to our knowledge, no study has explored what persons with MS and their formal care providers think of mHealth solutions for PA.

**Objective:**

The aim of this study was to (1) explore MS-specific needs for MS mHealth solutions for PA, (2) detect perceived obstacles and facilitators for mHealth solutions from persons with MS and health care professionals, and (3) understand the motivational aspects behind adoption of mHealth solutions for MS.

**Methods:**

A mixed-methods design study was conducted in Kliniken Valens, Switzerland, a clinic specializing in neurological rehabilitation. We explored persons with MS and health care professionals who work with them separately. The study had a qualitative part comprising focus groups and interviews, and a quantitative part with standardized tools such as satisfaction with life scale and electronic health (eHealth) literacy.

**Results:**

A total of 12 persons with relapsing-remitting MS and 12 health care professionals from different backgrounds participated in the study. Participants were well-educated with an even distribution between genders. Themes identified during analysis were MS-related barriers and facilitators, mHealth design considerations, and general motivational aspects. The insights generated were used to create MS personas for design purposes. Desired mHealth features were as follows: (1) activity tracking, (2) incentives for completing tasks and objectives, (3) customizable goal setting, (4) optional sociability, and (5) game-like attitude among others. Potential barriers to mHealth apps adoption were as follows: (1) rough on-boarding experiences, (2) lack of clear use benefits, and (3) disruption of the health care provider-patient relationship. Potential facilitators were identified: (1) endorsements from experts, (2) playfulness, and (3) tailored to specific persons with MS needs. A total of 4 MS personas were developed to provide designers and computer scientists means to help in the creation of future mHealth solutions for MS.

**Conclusions:**

mHealth solutions for increasing PA in persons with MS hold promise. Allowing for realistic goal setting and positive feedback, while minimizing usability burdens, seems to be critical for the adoption of such apps. Fatigue management is especially important in this population; more attention should be brought to this area.

## Introduction

### Background

Multiple sclerosis (MS) is one of the world’s most common neurologic disorders. MS is an unpredictable, often disabling disease of the central nervous system that can adversely affect body functions, and it is the leading cause of nontraumatic neurologic disability in young adults in many countries [1]. The most common symptoms are overwhelming fatigue, visual disturbances, altered sensation, cognitive problems, and difficulties with mobility [2]. There are pharmacological treatments for the condition as well as other strategies to manage MS symptoms. Quality of life is often impacted in many ways, and MS symptoms often lead to embarrassment and avoidance of social situations [3]. MS has a median survival time of around 40 years from the time of diagnosis [4]; therefore, issues regarding progressive physical and cognitive disability, psychosocial adjustment, and social reintegration are likely to affect persons with MS for a long time. Living with MS often requires individuals to self-manage and to be more engaged in their care [2]. Evidence suggests that physical activity (PA) helps people with MS stay active, reduces MS symptoms such as fatigue, and improves cognitive abilities but still many individuals with MS avoid PA [5-9]. Engaging individuals in specific behaviors involves understanding what motivates them to act in a certain way. Self-determination theory (SDT) is a macro theory of human motivation that establishes three psychological needs that motivate the self to initiate behavior and include the need for competence, autonomy, and psychological relatedness [10]. The implications of living with MS for patients, caregivers, treating clinicians, and society represent an opportunity for other modalities of care.

Connected health (CH) is a new model of health management in which patients become the center of the health care system with the support of new information and communications technologies (ICTs) [11]. The delivery of health care through mobile devices is known as mobile health (mHealth) [12] and is included in CH. The use of mobile software apps for health and well-being promotion has grown in recent years [13,14]. The use of mHealth for behavioral interventions has many potential advantages because of their ubiquity, cost-effectiveness, less invasive nature to participants, ability to provide immediate feedback, and track activities [15-17]. Persons with MS may benefit from the use of mHealth solutions supporting them in the management of their condition. However, to be effective, interventions need to reach the intended audience in a way that is meaningful to them. Condition-specific mHealth interventions require in-depth understanding of the patient and condition’s needs, barriers, and facilitators [18,19]. The process of tailoring refers to creating individualized communications by gathering and assessing personal data related to a given health outcome to determine the most appropriate strategy to meet patient's unique needs [20,21]. The important role that health care professionals have in the care of chronic patients is in contrast with their lack of involvement in mHealth apps development [22-27].

There are emerging trends in software development such as user-centered design (UCD) that try to address these problems, with the goal of creating solutions specific to the characteristics and tasks of the intended users [28]. Following UCD design principles generates systems that are easy to learn, have higher user acceptance and satisfaction, and lower user errors. UCD involves end users and relevant stakeholders in the different phases of software development process [28-30]. Access to mHealth end users, however, is not always easy or cost-effective; so, user representations such as personas are sometimes used. Personas are a common tool used in UCD to represent a target population and are created using information obtained through interviews, focus groups, and demographic data among others. These personas typically comprehend short descriptions that include the behavioral patterns, goals, skills, and attitudes of these user types [30]. Personas can be role-played to act as a vehicle to communicate user needs and requests to the designers and developers. Having personas helps designers focus on the users’ needs in a more concrete way, so that they can center their design on them.

Research in MS so far has focused on various health-promoting behaviors rather than specifically on PA [31-35]. In our preliminary study of commercially available MS mHealth apps [27], we encountered only a handful of apps (n=25), which is in stark contrast with the reality for other conditions such as cancer (n=295 in 2013) [36], diabetes (n=137 in 2009) [37], or human immunodeficiency virus (n=124 in 2013) [38] among others. To our knowledge, no study has explored what perspectives persons with MS and their formal caregivers have with regard to using mHealth solutions for PA.

To address the gap in the literature, we conducted a mixed-methods research with the goal of understanding the potential benefits of mHealth in individuals living with MS from two perspectives: the patient side (persons with MS) and the health care provider (HP) side (those professionals who work with them).

### Objectives

The aim of our study was to (1) explore MS-specific needs for MS mHealth solutions for PA, (2) detect perceived obstacles and facilitators for such mHealth solutions from persons with MS and health care professionals, and (3) understand motivational aspects that could facilitate development of mHealth solutions for MS.

## Methods

### Study Design

This study adopted a mixed-methods design: a qualitative part comprising focus groups and interviews, and a quantitative part comprising structured surveys and standardized tools.

Qualitative inquiries are useful to provide insight into complex and multifaceted experiences of individuals when a rich description is the main goal of the study [39]. On the patient side, focus groups and individual interview sessions were conducted to gather information on their use of ICT, health literacy, perceived obstacles and facilitators for PA and the use of mHealth solutions, and possible motivational aspects. On the HP side, focus groups’ individual interview sessions were conducted to explore what in their expert opinions are barriers and facilitators that could help patients with MS adopt healthier behaviors and what elements should mHealth solutions feature to be of use for patients with MS and health care professionals.

The quantitative part consisted of demographic questionnaires, satisfaction with life scale (SWLS) assessments [40], measurements of electronic health (eHealth) literacy (eHEALS) [41], and questionnaires on technology use. These quantitative assessments were used to contextualize the results obtained from the qualitative methods.

### Setting

Kliniken Valens is a center specialized in neurological rehabilitation services located in Valens, Switzerland. Kliniken Valens employs a multidisciplinary staff, including neurologists and physio-, occupational, speech, and sports therapists. In 2016, a total of 2451 patients with neurological conditions were admitted for neurological rehabilitation, of which 586 suffered from MS.

### Recruitment

Persons with MS from Kliniken Valens patient database were invited to participate in the study. Inclusion criteria required that each participant should (1) be older than 18 years, (2) have been diagnosed with MS, (3) have none to moderate physical disability (Expanded Disability Status Scale [EDSS]<4.5) at the time of recruiting, and (4) ownership and usage of a mobile phone. Participants were coded as PWMS from 01 to 12, that is, *PWMS01*.

For the HP side, physicians, physio-, occupational, and sports therapists who worked at Kliniken Valens were detected. Inclusion criteria were as follows: (1) be older than 18 years (2) have been working with persons with MS for more than 2 years, and (3) be a mobile phone user. Participants were coded as HP from 01 to 12, that is, *HP01*.

To ensure that the sample was rich for analysis, purposive sampling was used. The sampling was based on several factors such as EDSS scores, age group, and ICT familiarity for persons with MS; health care profession and years of experience, among other factors, were considered for HPs. Recruitment continued until saturation of results was reached.

### Ethical Approval and Informed Consent

Ethical approval for this study was obtained from the Swiss Ethics Committee on Research Involving Humans ID #2016-00529. Before agreeing to participate, all subjects were informed about the nature of the research project; the reasons for their subjectability; risks, benefits, and alternatives associated with the research; and their rights as research subjects.

### Data Collection

We used a semi-structured approach led by facilitators with experience in qualitative research providing trigger questions to participants; initially, questions were more general and gradually became more specific. The facilitators were GG and JK, physician and physiotherapist, respectively, who were present in all sessions. The questions derived from relevant points in the literature and UCD techniques [42]. See Multimedia Appendix 1 for guiding questions.

As the study progressed, emerging issues were explored with subsequent participants to refine categories and themes. Focus groups and interviews were conducted in German and English; German transcripts were later translated to English. Translated transcripts were linguistically and culturally validated through back-translation techniques and evaluated by bilingual professional translators.

The eHEALS scale attempts to determine a person’s combined knowledge, confidence, and perceived skills in finding, evaluating, and applying electronic health information to health problems [41]. The measure consists of 8 items scored on a 5-point Likert scale ranging from 1 (strongly disagree) to 5 (strongly agree). Higher scores on the eHEALS indicates higher eHealth literacy (total score range: 5-40). The SWLS is intended to represent a broad, reflective appraisal of a person’s life as a whole without differentiating between different domains [40]. This measure consists of 5 items scored on a 7-point Likert scale anchored by the extent of agreement with each statement. Items of the SWLS are summed to create a total score that can range from 5 to 35. Culturally and linguistically validated versions of the SWLS [43] and eHEALS [44] tools were used in this study.

### Data Analysis

Focus groups and interviews were audiotaped, transcribed verbatim, and coded using the qualitative data analysis management program NVivo (QSR International, Melbourne, Australia). Data analysis was conducted by 2 reviewers independently (GG and OR). Through iterative process, recurring themes and subthemes were identified and coded. During a deductive phase, coders matched each participant’s comment categorizing them as barriers or facilitators. An inductive phase came later where thematic content analysis was performed [45]. Further refinement was conducted by merging and removing redundant themes until consensus was reached.

The results of the standard structured questionnaires were analyzed according to their respective evaluation matrices.

### Persona Creation

To create personas that could work as intermediate constructs in the task of designing mHealth solutions for persons with MS, we used the information and insights generated in this study. The research team revisited observation notes, interviews and focus transcripts, and survey responses to define specific characteristics of the study participants and generate profiles. The initial profiles were refined and reviewed to generate personas as seen in other studies [46,47]. Additional characteristics such as stories were incorporated for further understanding of a user representation. Personas were then validated by HPs with experience treating persons with MS. With these personas in mind, specific strategies or tools can be created that fit the needs, goals, and tasks of these individuals.

## Results

For the patient side, we conducted 3 focus groups with 10 participants in total and 2 individual interviews. For the HP side, 2 focus groups with 8 participants in total and 4 individual interviews were conducted.

### Participant Characteristics

Table 1 provides a summary of participant characteristics for this study for the patient side (persons with MS). The patient side ages ranged from 35 to 62 years, with a median of 43.5 years (interquartile range [IQR] 40.25-50). Participants were well educated with an even distribution between genders. In terms of eHealth literacy, according to the eHEALS scale, the median score was 17.75 (IQR 11-28.50). The most common type of MS present was relapsing-remitting multiple sclerosis (RRMS), and the patients were being treated with immunomodulators. Participants had been living with MS for a median of 17 years (IQR 10.50-21.50), and according to the SWLS, most participants were dissatisfied with their lives (SWLS<14 [IQR 9-14]).

In Table 2, we can see characteristics of the HPs. In addition, ages of the HPs ranged from 26 to 64 years with a median of 40 years (IQR 28-53.25), and genders were equally distributed. The median of years of experience dealing with persons with MS was over 15 years (IQR 4.50-23). ICT ownership and use were very high in this group.

**Figure 1:**
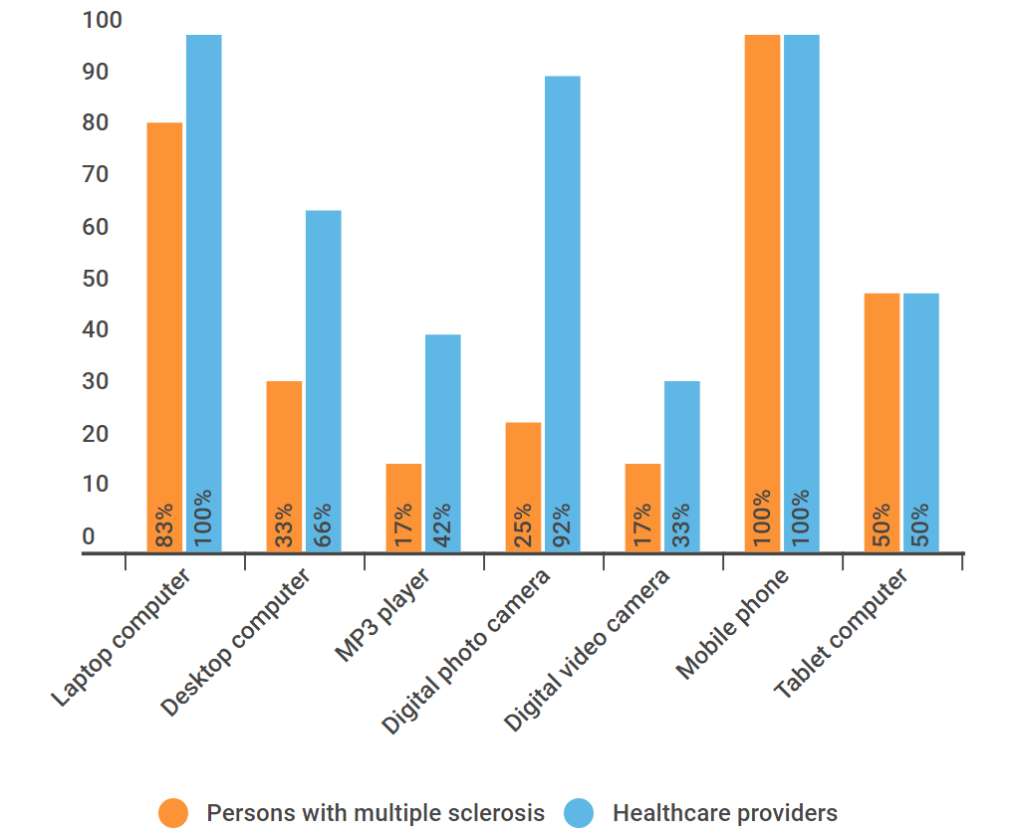
Information and communications technologies ownership.

**Figure 2:**
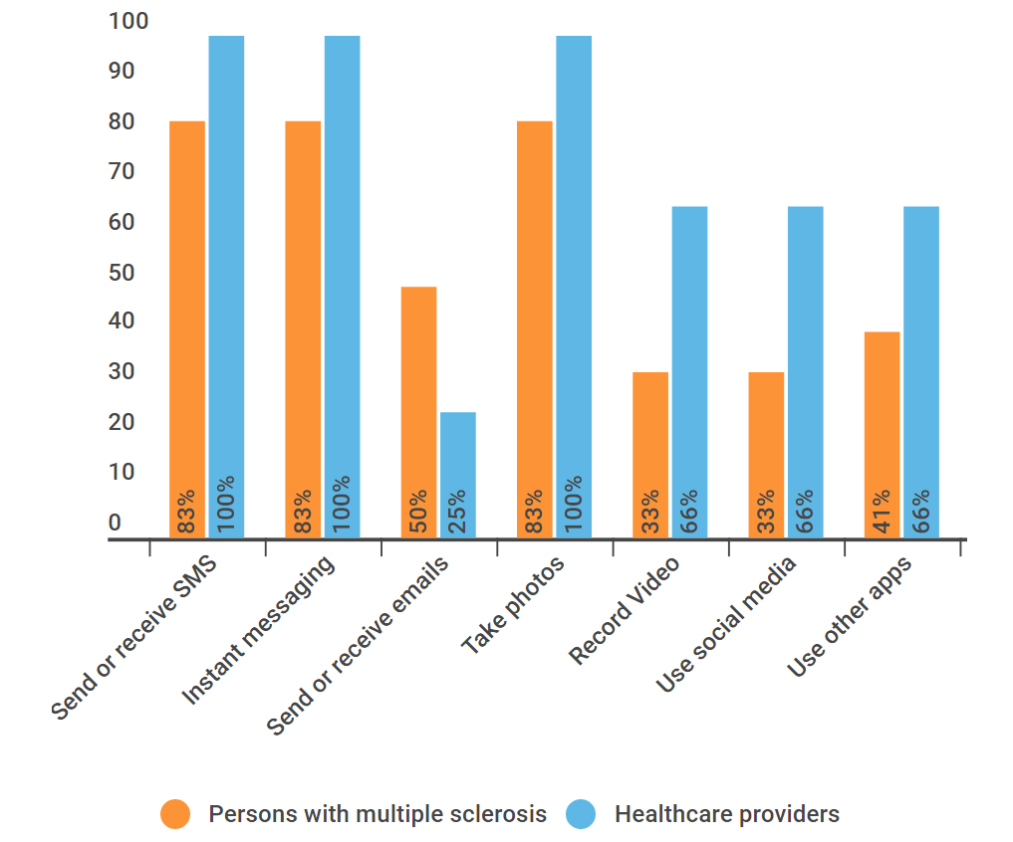
Mobile phone usage. SMS: short message service.

Ownership of ICTs was high as most individuals had laptops, desktops, and mobile phones and were frequent users of mobile phones (Figures 1 and 2).

### Thematic Analysis

Certain themes were identified during analysis: MS-related barriers and facilitators, mHealth design considerations, and general motivational aspects. Subthemes were also found and are presented in this study. Each theme and subtheme are presented mainly from the perspective of patient and bringing the HPs’ side to either reinforce or contrast relevant points.

A general overview of all barriers and facilitators to PA for persons with MS can be found in Textboxes 1 and 2.

#### Multiple Sclerosis–Related Barriers and Facilitators

To understand which, if any, specific MS barriers and facilitators there are to PA, we discussed general attitudes toward PA and how they coped with living with their condition. This produced certain subthemes:

##### Specific to Physical Activity

An important deterrent of PA was the diminishing sense of self-efficacy and the impact MS symptoms directly have in the enjoyment of PA. According to *PWMS02*, there are times when:

You don’t know how much confidence to have in yourself.

I used to do a lot of sports. 80 km of jogging a week, tennis, cross-country...Over time, it became less and less. My motivation has decreased because of MS. I still enjoy it, but not quite like I used to. Now, it feels like work.

PWMS09

HPs own assessments of the situation were in agreement:

Since everything requires exertion, the fun factor and enjoyment are missing somehow, so why [should they] do it?

HP08

The need for goal-setting and proper feedback was deeply emphasized in this part of the conversation. Being able to understand when progress is being achieved was considered key as the subjective experiences differed from what they actually accomplished:

[In general, if you want] to convince people that physical activity is the key, we need to give them targets. Having feedback to how you are doing is good. We need to know we are doing something right.

PWMS06

If you ask them, “how do you feel,” they will always say, “I don't feel good.” Interestingly, this feeling doesn’t change, they may train over 3, 4, or 5 weeks and they will feel the same. However, if you look at the parameters that you normally assess, you will see that they have improved. VO_2_, oxygen uptake, or maximum heart rate will have gone up. They objectively improve but subjectively still feel bad.

HP11

The important thing is that we have to show [them] clear goals. These goals have to be realistic, measurable, and achievable. [...] you have to work toward that step by step.

HP10

Persons with MS and HPs were in agreement: customizing PA to meet a patient’s individual need determines the success or failure of an exercise program. Flexibility and engagement are required.

##### Fatigue Management

Fatigue and fatigue management issues were raised over and over again. Persons with MS reported that, as they went along their activities of daily life, they *accumulated* more and more fatigue. In this way, one participant stated that, "Fatigue ate away their life."

Participants claimed that they had to resort to “strange strategies” to be able to keep up:

I use one trick, I move all my appointments to the morning; so, people around me don’t realize that I’m not well. I then take a break in the afternoon, and if someone wants to do something, I just say that my calendar will free up again in the evening.

PWMS02

**Table 1:** Participant characteristics: persons with multiple sclerosis.

Characteristics		Persons with MS^a^ (n=12)
**Gender, n (%)**		
	Female	6 (50)
Age (median, IQR^b^)		43.5 (40.25-50)
**Education^c^** **, n (%)**		
	High school	2 (17)
	Higher education	6 (50)
	University or college	4 (33)
**Marital status, n (%)**		
	Single	2 (17)
	Married	8 (66)
	Divorced	2 (17)
**Employment status, n (%)**		
	Not working	1 (8)
	Unable to work	2 (17)
	Employed	9 (75)
**Type of MS, n (%)**		
	Relapsing-remitting MS	7 (58)
	Secondary-progressive MS	3 (25)
	Primary-progressive MS	2 (17)
	Progressive-relapsing MS	—
Years since MS diagnosis (median, IQR)		17 (10.50-21.50)
EDSS^d^ score (median, IQR)		4 (3.75-5.12)
**Pharmacological treatments, n (%)**		
	Immunomodulators	7 (58)
	Muscle relaxants	4 (33)
	Antidepressants	3 (25)
	Vitamin supplements	5 (42)
	None	2 (17)
SWLS^e^ score (median, IQR)		12 (9-14)

^a^MS: multiple sclerosis.

^b^IQR: interquartile range.

^c^Categories were simplified from the Swiss Education System.

^d^EDSS: Expanded Disability Status Scale.

^e^SWLS: satisfaction with life scale.

**Table 2:** Participant characteristics: health care providers.

Characteristics		Health care providers (n=12)
**Gender, n (%)**		
	Female	6 (50)
Age (median, IQR^a^)		40 (28-53.25)
**Health care profession, n (%)**		
	Physiotherapists	6 (50)
	Occupational therapists	2 (17)
	Sport therapists	1 (8)
	Physicians	3 (25)
Years of experience (median, IQR)		15.5 (4.50-23)

^a^IQR: interquartile range.

**Overview of barriers for physical activity in persons with multiple sclerosis.**

Motivational aspects

- Social comparison with others with multiple sclerosis
- Negative feedback from their environment
- Self-motivation issues

Multiple sclerosis symptom burden

Balance problems

- Muscle weakness and fatigue
- Bladder control
- Medication side effects
- Unpredictable course

Physical activity misconceptions

- Fear of triggering a relapse
- Poor understanding of benefits
- Unrealistic expectations

**Overview of facilitators for physical activity in persons with multiple sclerosis.**

Motivational aspects

- Social support from loved ones
- Collaboration with other persons with multiple sclerosis
- Clear goals
- Reminders from third parties

Physical activity promoters

- Reserved dedicated time for physical activity
- Personalized training routines
- Sufficient recovery time
- Noticeable benefits

HPs commented that sometimes it is helpful for persons with MS to create some sort of visual representation of the body’s energy sources. *HP07* related this with patients’ difficulty *determining how much energy they will need for [doing] something*. According to *HP05*, resources are limited in patients with MS; so, they have to learn how to manage them. Another HP stated:

If we had an app that would allow patients to manage their energy as a resource, [now] this would be a great thing.

HP10

When asked about what they would feel about such a “fatigue management solution,” patients responded very positively:

That would be awesome, yes, definitively. If there would be something that would measure how much energy I have left for the day and how much I’ve already used so far. That would be excellent. It would be amazing.

PWMS06

##### Living With Multiple Sclerosis

MS conditioned the way persons with MS live their life; not only in that they have to consider their energy as resources that need managing but also in more subtle ways. Many persons with MS report that weather conditions and warmer temperatures worsens their symptoms; *PWMS07* stated: "Heat makes me sluggish."

Some experience bladder and bowel problems that shape how they plan their daily routine.

The progressiveness of the disease acts as a strong barrier and reduces motivation for acquiring healthier behaviors. One participant stated:

I don’t want to change the way [I live my life] because a relapse may happen and then what [was the point of changing them]?

MS gets in the way of doing things

PWMS02

*HP03* suggests that this is because:

They don’t want to grapple with the disease and just want to do things like other people of their age, […] patients often struggle with themselves and give the disease very little room [in their life].

*HP03* recommends professionals who wish to work with persons with MS to pay special attention and make an extra effort to understand the psychological distress they may experience:

MS isn’t always easy to understand for us. MS patients are more sensitive because of the condition, one wrong word can be enough to demotivate them.

Both sides felt that persons with MS would benefit more if they had some form of cognitive activity they could do to stimulate them and prevent further deterioration.

#### mHealth Design Considerations

The following were design considerations of interest to designers of mHealth solutions. They identify barriers to adapt design approaches. Although the exploration was focused on mHealth solutions, it was common for participants to use the word “app” interchangeably; this change in terminology has been kept intact when quoting participant’s views. A summary of suggested mHealth solution features and characteristics that emerged from our interactions is presented in Textbox 3 in order of feature priority. An overview of barriers and facilitators can be found in Textboxes 4 and 5.

##### Attitudes Toward mHealth

On this topic, both sides were interested but hesitant. The main concern was regarding the value an mHealth solution could hold. ICT usage barriers were mentioned as *those who are not interested in technology would never use an app anyway*.

**Potential features and characteristics for multiple sclerosis mobile health (mHealth) solutions.**

Customizable goal setting

- Challenges need to be tailored to the specific person with multiple sclerosis characteristics

Energy profiles and fatigue management

- Information and tools that help users in managing their day-to-day activities

Patient education

- Offer verified information that is helpful and reliable

Data visualization

- Information must be presented in a way that is meaningful to persons with multiple sclerosis

Positive feedback system

- Rewards and incentives for completing tasks and objectives

Activity tracking

- Register metrics such as steps, calorie consumption, heartbeat, and quality of sleep among others

Exercise library

- An array of different activities specific to multiple sclerosis such as fitness or relaxation techniques that can be selected

Game-like attitude

- Engaging in a playful mindset in a way that is highly pleasurable and motivating

Strong evidence base

- Features and information offered should have a solid scientific foundation

Remote monitoring

- Health care providers can follow persons with MS progress and give feedback

Optional sociability

- Ability to opt-out of social media features such as messaging, feeds, or other types of social comparisons

Reminders systems

- Notifications that reminds persons with MS to engage in activities

Personal data management

- Access to personal information and data defined by the user case by case

**Overview of barriers to the adoption of multiple sclerosis mobile health (mHealth) apps.**

Social Factors

- Negative word of mouth from peers or health care providers
- Disruption of the health care provider-patient relationship
- Promotes competition among multiple sclerosis peers

Reliability of the solution

- Unrealistic promises
- False information
- Inaccurate measurements

User experience

- Rough on-boarding experience
- Obvious or excessive advertising
- Constant notifications or reminders

Usability

- Unattractive design
- Confusing interface
- Accessibility issues

Value proposition

- Solution does not fit the needs of users
- Unclear purpose
- Overall lack of personalization

Data ownership and access by third parties

Refusal to use information and communications technologies

When confronted with the question of whether they would use a mobile solution for MS, many were intrigued but unsure about *how an app would benefit* them:

The effectiveness isn’t clear to me.

PWMS01

That's what I can’t think of. What does the app give them?

HP11

It maybe true that we [health care professionals] are not likely to recommend or suggest technology-based solutions. I’ve never thought about it. Maybe because there is still no clear answer as to how apps can help. Perhaps, we feel that the personal relationship that we form with our patients is not something we can replace with technology.

HP05

Items that increased the intent of downloading and using mHealth solutions were knowing that experienced professionals were involved in the design and having endorsements from recognized MS institutions. A point where all HPs agreed on was that mHealth solutions for MS should be based on solid scientific information and theory. For health care professionals, it was a matter of tool validation, whereas for patients, it seemed to be more about effective word of mouth. The strongest motivators for downloading or recommending an mHealth solution were clarity in its features and promises, and solid scientific backing:

[I read] the description and what it offers [to me]. [I like it] if there are bullet points about what it will give me. Perhaps something like having a manual about how to use it. […] I think that’s something that I look for before installing.

PWMS06

If an app has theoretical basis behind it and it’s useful for the patient, I would feel comfortable [recommending it]. Even if it doesn’t have publications [proving it works].

HP05

The main deterrents for installing, and most influential factors preventing HP recommendation of an mHealth solution, were the presence of false information and negative experiences from acquaintances or read on the news.

**Overview of facilitators to the adoption of multiple sclerosis mobile health (mHealth) apps.**

Social factors

- Endorsements from experts and patient associations
- Reinforces the health care provider-patient relationship
- Allows collaboration and support amongst multiple sclerosis peers
- Integration of family and friends in the solution use and flow

Reliability of the solution

- Up-to-date information
- Friendly language
- Theory and evidence based

User experience

- Customizable features
- Variety of options
- Playfulness

Usability

- Simple to use
- Attractive design
- Consistent interface

Value proposition

- Benefits of use must be evident
- Provides incentives and motivation
- Specific to persons with multiple sclerosis needs

Data ownership and access management

Designed and developed in collaboration with health care providers

A shared view among HPs was that these solutions should not get in the way of standard care, rather they should act as additional support tools that could let professionals guide patients from the distance:

An app can be used to motivate people. The app can be like a kind of coach. A virtual coach that gives them a task and if they do it, they’ve reached a partial goal, for example, [they get some incentive]. And they know how many points they earned by the end of the month. That could be an incentive.

HP08

This sentiment was in line with what persons with MS were expressing, for example:

[an app could present something like] an obstacle course that you have to get through. [Something] that you tackle daily. The app would have to give you an alert that says you have to walk 2 km today, for example. And you have to be able to set [your own] goals. The patient should try how long he or she can walk and then perhaps increase the amount. That would maybe make people use it more. In a game, there are also tasks that you have to do. If you finish them, you get something.

PWMS02

This game-like attitude heavily resonated in several other patients and even some HPs:

For me, it’s important that (the app) is playful. We all remain children deep down. It should have colors, some music and be attractive.

HP03

Personalization and customization was regarded highly in both groups, yet as *PWMS07* says it is important to remember that:

Everyone is as active as they want to be. The app is of no use if the person doesn’t want to do things.

##### eHealth and Health Literacy

Participants with MS held in high regard the opinion of their HPs, often consulting them for information validation or seeking advice. A common concern was not about finding information on the Web, but rather making sure that it was right for them.

There are a lot of types of MS and what may help one person might harm another one

PWMS06

HPs were reticent on directing their patients to any online sources:

They can find information online, so there’s no need for a special app for that I think. However, you can get lost in the sea of the Internet and you may need an expert to guide you.

HP10

The need for reliable information regarding other symptoms was mentioned:

We may need information about incontinence. What to do if your bladder cramps up? Maybe knowing about pelvic exercises [would be useful].

PWMS11

There was a lot of uncertainty about which activities would be beneficial and not harmful to them. Because of their condition, participants with MS feared engaging in new activities as these are “untested waters.” This was seen not only in terms of PA but also for nutrition:

I have equipment for training at home, but I don’t know if I use it correctly or at the right time. I want to exercise a group of muscles but I don’t know if that will hurt another group [of muscles]. What should I be eating now? I don’t know what to do.

PWMS07

The health care community seemed to be in part to blame for these anxious feelings. MS misconceptions and outdated knowledge among professionals played a role in fostering this uncertainty:

I have a doctor who tells me that I have to do less, as less as possible. […] Otherwise, I might do too much and put too much strain on my body, and this could possibly trigger a relapse.

PWMS02

Many neurologists are telling patients that they shouldn’t do much physical activity, or that they are not allowed to do some sports. [in the past] patients and medical reports have described a deterioration of symptoms due to PA and the main view was to not recommend training to avoid this deterioration. But now, we know that this is only a temporal setback, just for a few hours and then people recover completely. It has no lasting effect on MS symptoms; after a resting period, functions are restored to normal level. There is also no risk to induce a relapse. A recent study, published last year I think, shows that there is no correlation between physical training, even in higher intensities, and a risk of inducing a relapse, but not all of us [professionals] stay updated.

HP05

##### Privacy and Data Ownership

Participants with MS and HPs had negative perceptions of third party involvement in mHealth projects. For the patient side, the main objection was in terms of pharmaceutical or insurance companies taking advantage of their medical and personal data. They saw their participation in mHealth projects as some sort of a warning sign and expected their involvement to be explicitly clear upfront:

I’d like to know who’s getting the data and what for. It’s my personal data.

PWMS07

If everyone could see my data, I wouldn’t give [the app] a chance.

PWMS01

The HPs were less opposed to hearing about pharmaceutical companies being involved but still were concerned. HPs wondered whether it would be possible to restrict these companies from accessing sensible data:

I don’t want to have these [pharmaceutical] companies having access to that information. The commercial interest is dangerous in this way. I feel reluctant to give too much information […] to health insurance companies even. I think it’s an aspect that needs discussion and setting up clear rules for all participants.

HP05

#### General Motivational Aspects

Using SDT as lenses, we coded participants with MS’ comments and responses with regard to competence, autonomy, and relatedness.

##### Autonomy

Autonomy within SDT concerns a sense of volition or willingness when doing a task; events or conditions that diminish the sense of choice interfere with perceived autonomy. All participants with MS wanted to, in some degree, be able to influence their condition treatment. They wanted to be able to set their own goals or decide what activity to do at a given time. They wanted to feel that they have a choice in the matter. For example, *PWMS04* said he needs to find a way in which doing the task is his decision:

[I am doing it] not because I have to do it, but because I want to do it. And [only] then I can do it.

The loss of perceived autonomy seemed to play an important role. It was often mentioned as barrier and facilitator at the same time. It presented itself as a cause for concern and depression for some and as a motivator for others:

I can’t do everything I did before [I was diagnosed].

PWMS03

Self-motivation is very difficult. I always need something that I can’t do anymore and then I want to be able to do it again.

PWMS01

##### Competence

Competence refers to the need for challenge and feelings of effectance; opportunities to acquire new skills or to receive positive feedback increase perceived competence. For participants with MS, acknowledgment of their progress and tracking was very important:

At first, I could only walk 6 meters and now I can do 180 meters without taking a break. That makes me happy. I feel more like doing something.

PWMS03

Presenting situations as challenges to overcome was highly motivating for them, but there were some caveats. *PWMS06* stated:

I’d rather be amongst healthy people and have the challenge to keep up with them.

*PWMS07* remarked that in his case, he needed:

To surpass his limits every day but that it was important to understand that you shouldn’t be in competition with other persons with MS. We need to be supportive [to each other].

##### Relatedness

Relatedness is experienced when a person feels connected with others, positive social interactions enhance the feeling of relatedness. The way participants seemed to discuss social interactions required a clear distinction between how they engage with others with MS and with people without MS.

###### Others With Multiple Sclerosis

The relationship participants with MS have with others with MS is complex. Interacting with those who share their condition had a very strong negative impact as evidenced by comments such as:

I don’t want to speak to everyone who has the same disease. That doesn’t help me. If you get to talk with someone who shares the same values and goals, that's good. But if you wear glasses, you don’t want to speak with everyone just because they wear glasses too.

PWMS01

It’s very depressing. It doesn’t really help me [seeing others with MS], let’s put it this way. People with MS tell me "Oh, you can still do this, I can’t anymore” or "Oh, I can’t sleep because everything hurts." They tell me that they are always exhausted and are always tired. It just drains me [to hear them]. It takes away all my energy.

PWMS06

I always saw other patients and heard many bad stories. I feel that having a negative or positive attitude is what determines things. I once received a request for a forum where you sit in a circle and talk with other MS patients. I don’t need such a self-help group.

PWMS08

This particular aspect was very much so present for the HPs as they noticed that:

There are patients who tell us that they don’t want to see other patients with MS [with more advanced MS than them] because they don’t want to see their future.

HP12

However, spending time with other people with MS was not always a negative thing. Going through the same experience provides a common ground that they share:

I’m in a regional [MS] group. We go on excursions or meet for coffee. Then, we talk about everything but the disease.

PWMS05

I don’t stress when I’m with them [persons with MS], [I don’t think] about the weakness in my legs or [the way I look with] my walking. I know that they experience the same problems that I do or worse; so, it takes some of the stress out because I don’t feel like I’m being watched.

PWMS06

It’s important to distinguish how you’re connected. I don’t want to compete [with other persons with MS].

PWMS07

It’s important to do things in a group. It’s much better than being alone. The motivation is stronger that way.

PWMS09

###### People Without Multiple Sclerosis

Having a social circle of family and friends who provide support was a determining factor for motivating persons with MS to take better care of themselves. This was present in all interviews and participants. HPs had a slightly different take on this, as family members’ expectations can have their downsides:

If the partner is healthy, they [persons with MS] often put themselves under too much pressure [to perform].

HP08

The way strangers look at them had a big effect on participants with MS, to the point that some of them try not to move just to limit what can be seen and criticized:

If I’m not having a good day, I won’t leave the house. I’d know early on in the morning. [I know that] I’ll have balance problems…I've been told a few times by strangers that “I should drink less.” If I’m really having a bad [symptoms] day and someone comes and says something like that, it gets to me.

PWMS10

However, friends and family remind them that:

We’re not alone with our MS. There are people thinking about what they can do to help us.

PWMS08

### Persona Creation

The information collected from the questionnaires, structured surveys, focus group, and individual interviews was reviewed and used to devise case profiles. We identified specific characteristics of our participants, such as age, level of PA, ICT usage, and general motivations, and used clustering to create 4 MS persona types: (1) high ICT, medium PA; (2) medium ICT, high PA; (3) medium ICT, medium PA; and (4) low ICT, low PA.

Personas created represent potential persons with MS and highlight their individual barriers and facilitators to mHealth adoption:

- Demographic information from our participants was used to generate socioeconomic traits.
- Medical information from the participants was summarized and grouped to create medical profiles.
- eHEALS scores were converted to persona traits that represented the eHealth and health literacy levels.
- SWLS and interviews helped define personality traits such as a life perspectives or sociability.
- Data from the focus groups and interviews helped create the different stories.

**Figure 3:**
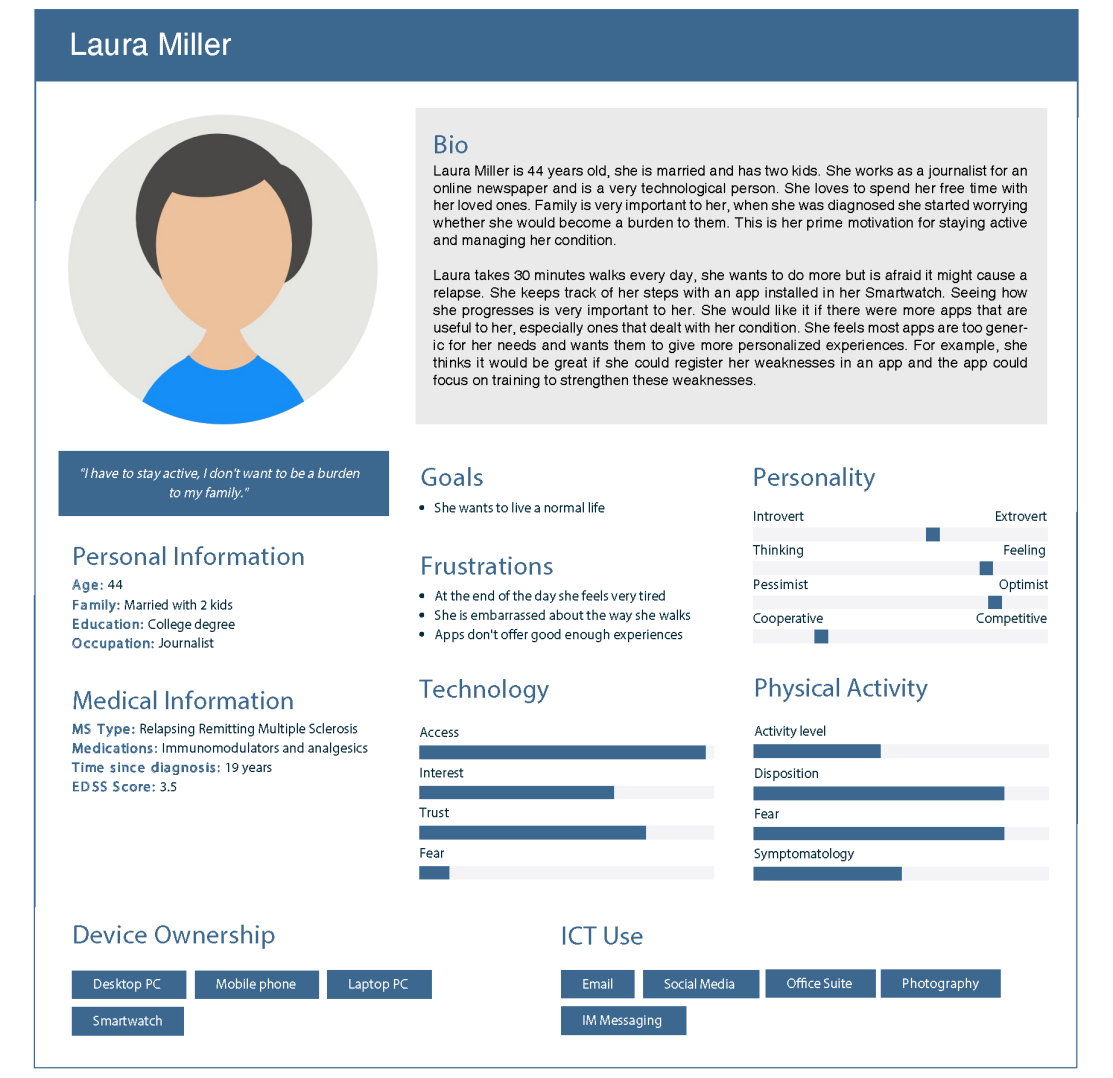
Multiple sclerosis persona with high information and communications technologies and medium physical activity.

Figure 3 shows an example of our MS personas. See Table 3 for a synopsis of all MS personas; full versions can be found in Multimedia Appendix 2.

Table 3 presents a summarized version of the MS personas; for the full version, please see Multimedia Appendix 2.

**Table 3:** Multiple sclerosis personas’ synopsis.

Persona name	Age	Gender	Years with MS^a^	Type	Description
Laura Miller	44	Female	19	High ICT^b^, medium PA^c^	Laura is married with 2 kids, and her family is very important to her. She is afraid that MS will make her a burden to those around her. She understands she should work out more but feels tired all the time. She likes to use technology and has a smartwatch.
Tim Smith	42	Male	5	Medium ICT, high PA	Tim is an elementary school teacher. He is married to Margaret who always wants to go for walks with him. He likes to stay positive so he tried not to talk about MS. He likes using step counters because he feels he achieves things that way.
John Peterson	38	Male	12	Medium ICT, medium PA	John is an office clerk and not a big fan of technology. Things are complicated enough as they are. He has been having problems with his eyesight. He does not work out because he is afraid to trigger a relapse. His wife tells him that he gets too competitive sometimes.
Amanda Palmer	47	Female	15	Low ICT, low PA	Amanda is divorced and does not like to exercise. She does housework and feels that is enough. She does not really understand technology or why people would use it except for the basics. Friends are very important to her.

^a^MS: multiple sclerosis

^b^ICT: Information and Communications Technologies

^c^PA: physical activity

## Discussion

### Principal Findings

We conducted a series of focus groups and interviews with persons with MS and health care professionals in charge of their care, and identified specific needs and characteristics for mHealth solutions. We also identified possible obstacles and facilitators for mHealth adoption. To our knowledge, this is the first study to bridge this gap in the literature. We analyzed four overarching themes (MS-related barriers and facilitators, mHealth design considerations, and general motivational aspects) with their respective subthemes. Important findings from this study include the identification of desired features in mHealth solutions for persons with MS such as: (1) activity tracking, (2) incentives for completing tasks and objectives, (3) customizable goal setting, (4) optional sociability, and (5) game-like attitude among others (Textboxes 4 and 5). Potential barriers to MS mHealth adoption such as rough on-boarding experiences, lack of clear use benefits, and disruption of the HP-patient relationship are identified; potential facilitators were also identified such as: (1) endorsements from experts, (2) playfulness, and (3) tailored to specific persons with MS needs (Textboxes 4 and 5). We also explored barriers and facilitators for PA in persons with MS (Textbox 3). Lastly, we used this understanding to develop a set of personas that represent male and female versions of persons with MS, to provide designers additional means to help in the creation of mHealth solutions for MS.

### Comparison With Prior Work

#### Physical Activity and Fatigue

Only a small proportion of individuals with MS report meeting the minimum guidelines for PA for patients with MS [7,8]. PA and exercise have been the subject of much discussion in the MS literature, with attention to engaging patients in health behaviors aimed at reducing their physical limitation and improve their overall health and well-being [48]. However, persons with MS have a different attitude toward PA [49] and are typically less active compared with healthy persons [50]. This was also the case in our study, as patients expressed how working out now entails a new range of obstacles. The overall fear of triggering a relapse and further harm themselves was very much present. The outdated belief that “exercise is dangerous for patients with MS” has been demonstrated as incorrect, as symptoms’ impairment after exercise is only temporary and does not affect the disease course [51]. However, this continues to stop physical exercise prescription [52]. Findings also remark the importance of realistic goal setting and feedback on achieving progress, which is consistent with a meta-analysis on the effectiveness of setting goals for health [53]. The most common facilitator for PA was adjusting the type of exercise modality and intensity to the individual; this is in line with what has been called “appropriate exercise for physical capabilities” [54]. Accommodating for preferences, allowing persons with MS to select from a variety of activities may help foster autonomy and increase their enjoyment of PA. Several studies suggest that providing participants with opportunities to set priorities in choosing which health behaviors to focus on result in better outcomes [55,56].

The general lack of enjoyment of PA was a big demotivator for persons with MS. Including game elements or a game-like feel to PA was seen as positive and a desirable feature. Gamification is often defined as “the use of game design elements in nongame contexts” [57]. The use of gamification and serious games is a popular strategy in mHealth [58]; it would be interesting to explore its effectiveness in this population. However, as competition with others was viewed negatively, game features should be implemented with care to avoid mechanics that could be adversely received in this population.

Fatigue is a subjective sensation, with objective changes in mental or physical performance conceptualized as fatigability [59]. It was perceived as both an important adverse consequence of PA and a barrier to PA. Fatigue is typically worst for patients with MS in the later part of the day [60,61] and is exacerbated by psychosocial stress [62]; this phenomenon was experienced by several of our participants. An interesting point, frequently remarked on by HPs and persons with MS during this study, was the need for enforcing strategic “energy” management, which could be supported by ways to visualize “energy” expenditure.

#### mHealth Considerations

Health literacy is the degree to which an individual has the capacity to obtain, communicate, process, and understand basic health information and services to make appropriate health decisions [63]. Nowadays, health information also includes electronic resources such as the Internet and other technologies that now play an increasing role in consumer health [41]. Studies show that a prior use is the most important predictor of accepting new media for communication with HPs [64]. Participants in our group had already a widespread adoption of new communication technologies (computers, websites, emails, and mobile phones). MS online information sources are reported to have variable quality [65]. As with most long-term conditions, persons with MS information-searching habits vary depending on the time since diagnosis. Information needs vary along the course of the condition [66]. Persons with MS in this study valued their lead physician’s opinion above information found online. Official “professional endorsement” was high on their list of priorities for accepting online health information or mHealth solutions.

The idea of mHealth solutions for MS management was positively received; however, our preliminary study showed that there are very few mHealth solutions for persons with MS currently available [27]. The deciding factor for mHealth adoption seemed to be having a clear value proposition. Persons with MS held pleasant user experience in high regard to their engagement with mobile apps; apps should be simple and intuitive to use, which aligns with Nielsen’s findings on usability [67]. HPs felt that having theoretical background was essential. User privacy and ownership of user-generated data remains an underexplored territory from policy and regulatory perspectives [68]. HPs and persons with MS were concerned about data confidentiality, and how the use of mHealth solutions could impact on the doctor-patient relationship; this is in line with other findings in the literature [24,69-73].

Persons with MS are known to modify their social relationships and free-time activities as a result of their diagnosis, switching from group activities to individual exercises, resulting in worsening of their social life [49]. Feelings of frustration and loss of control may be the most commonly experienced self-evaluative negative consequence from participation in PA in persons with MS. Engaging with others with MS was easier for participants with MS because they felt less conscious about their limitations; however, it also served as a reminder of the uncertain progression of the condition. Most participants preferred to avoid discussion of MS and staying away from health-related topics. This aversion should be kept in mind when designing ICT interventions that include socialization features.

Designers of mHealth solutions for MS should also take into account condition-specific disabilities, such as reduced fine motor skills or blurry vision, to increase the chances of adoption. Besides having solid scientific content, these apps need to be designed to consider individual needs. The list found in Textboxes 4 and 5 of suggested mHealth solutions features provides an interesting starting point for exploration. It would be beneficial for future MS mHealth designers and developers to have the facilitators and barriers presented in Textboxes 4 and 5 in mind during the creation of new MS mHealth interventions.

The use of personas is relatively new in the field of mHealth and is being used to support the development process of health information technologies [28]. The MS personas we created and provided here (Table 3 and Appendix 2) can be used to guide designers in the creation of mHealth solutions but should be used considering their limitations.

### Limitations

This study has limitations that are inherent to qualitative research methods. The sample size is not large enough to be representative of a larger population. A potential limitation of this study is the recruitment method, as participants came from a single center from a highly developed country such as Switzerland; steps were taken to make the sample as diverse as possible, but the risk of selection bias is present. The level of education, economic status, etc, will surely be different in a sample from a different center in another country. HPs had different backgrounds, so, the resulting views represent an interdisciplinary perspective and not those of a single discipline in particular. Likewise, MS treatment and care services available vary depending on the country; so, persons with MS will have different views on how to manage their condition.

Exploring persons with MS mHealth needs is difficult as the distinction between “user needs” and “user wants” is not clear. This should be taken into account as interviewed subjects may inadvertently respond “needs” questions with their “wants.” Also, MS unpredictable progression influences the generalizability of this study, as the experiences differ from patient to patient; however, this limitation is inherent to MS.

Personas generated in this study need to be contextualized as coming from a high-income country and not addressing younger adults with MS; their use is limited and would require refining to better suit other populations.

Finally, our findings may be limited by the fact that the majority of participants were enrolled in a PA rehabilitation treatment plan from the clinic; so, their awareness to PA benefits may be positively biased.

### Conclusions

mHealth solutions have been advocated as a modality with the potential to increase efficiency within medical practice, and their use for increasing PA in persons with MS holds promise. Critical issues to address for an improved adoption of MS health solutions seem to be allowing users realistic goal setting, providing them with positive feedback, and minimizing usability burdens. Fatigue management is especially important in this population; more attention should be brought to this area. Results of this study provide valuable information that could help designers and developers of mHealth solutions for MS. It would be advisable that future mHealth interventions for MS consider the facilitators and barriers highlighted in this study. We are currently exploring how commercially available MS health apps contrast the findings of this study, aiming to understand how the current supply meets the demand. The combination of persons with MS-positive predisposition for specialized solutions for MS and the gap in mHealth solutions provides an interesting opportunity to explore. In the words of *PWMS10*: "There aren’t many apps yet for MS; so, it’s time to make an app."

## Appendix

Multimedia Appendix 1 Question guide.

Multimedia Appendix 2 Multiple sclerosis (MS) personas.

## Abbreviations

EDSS: Expanded Disability Status Scale
eHEALS: eHealth Literacy Scale
eHealth: electronic health
HP: health care provider
ICT: information and communications technologies
IQR: interquartile range
mHealth: mobile health
MS: multiple sclerosis
PA: physical activity
PWMS: person with multiple sclerosis
RRMS: relapsing-remitting multiple sclerosis
SDT: self-determination theory
SWLS: satisfaction with life scale
UCD: user-centered design
