# Supplementary material for: Exploring the Specific Needs of Persons with Multiple Sclerosis for mHealth Solutions for Physical Activity: Mixed-Methods Study
Source: JMIR Mhealth Uhealth. 2018 Feb 9;6(2):e37. doi: 10.2196/mhealth.8996 (PMC5889817; doi:10.2196/mhealth.8996)
Supplement: Multimedia Appendix 1 [file mhealth_v6i2e37_app1.pdf]

Appendix I. Question guides

Interview guide for persons with MS

Introductory question

During the first part of this discussion I would like to hear from you what you have done during the past year to increase your physical activity. What kind of things do you do to be physically active?

Guiding questions (by numbered topic)

1. Perceived obstacles and facilitators for physical activity

    a. What are the main issues you face when doing physical activity?

    b. What is the hardest part about being physically active?

    c. Is there anything in particular that makes you want to stop exercising forever?

    d. What sort of physical activity do you enjoy the most? (yoga, running, etc)

    e. Why do you think you don’t exercise regularly?

    f. Do you feel that your medical treatment impacts your ability to do physical activity in any way?

    g. What opportunities are in place for physical activity in your daily life?

    h. Are there times of the day where you feel you might be more inclined to do physical activity?

    i. How clear are healthcare providers about the need of physical activity with your condition?

2. Perceived obstacles and facilitators for use of mHealth solutions

    a. How do you feel about using an app to manage your condition?

    b. Do you think an app is likely to help you exercise more? Why?

    c. What aspects of your physical activity routine do you think could be improved by a mobile app?

    d. Are you concerned about who develops the apps you use? Who do you think should be involved in developing an app for your health?

    e. What makes you install an app? What makes you uninstall an app?

    f. What would you say is the main reason why you stop using an app for health?

g. In terms of user interface (the way an app looks and feels), what is something you value the most? What do you dislike the most?

h. Let's say that you can have your perfect app to help you be more physically active, what does this app do?

i. What sort of game aspects or game-like features do you enjoy the most?

3. Possible motivational aspects

a. Do your loved ones help you being physically active?

b. Is there any trick that you use to convince you to do things that you don't like doing?

c. How do you think you would act if you were presented with a physical activity routine that's personalized for you and your condition?

d. What strategies would you suggest to increase physical activity to a fellow person with MS?

e. Would being reminded during your daily routine that you need to be more physically active help you?

f. Do you think you'd feel more motivated to exercise if you knew more about the benefits for your condition?

g. How would you feel about being part of a group of people with MS who exercise regularly?

h. Would you feel more comfortable exercising in the company of other persons with MS?

i. Have you ever felt embarrassed of doing a physical activity? Has this embarrassment ever stopped you from doing it?

Concluding question

Of all the things we've discussed today, what would you say are the most important issues related to developing health technology to improve physical activity

Interview guide for health professionals

Introductory question

During the first part of this discussion I would like to hear what are common complaints that you hear from persons with MS about being physically active.

Guiding questions (by numbered topic)

1. Perceived obstacles and facilitators for physical activity

a. What is the hardest part about engaging patients with MS to be physically active?

   b. Is there anything in particular that you've noticed makes them stop exercising?
   c. Why do you think they don't exercise regularly?
   d. What sort of physical activity would you recommend to them?
   e. How do you express the need for physical activity to patients with MS?

2. Perceived obstacles and facilitators for use of mHealth solutions
   a. How would you feel about a patient with MS using an app to manage their condition?
   b. Do you think an app is likely to help them exercise more? Why?
   c. What aspects of a physical activity routine do you think could be improved by a mobile app?
   d. What makes you recommend a health app? What makes you warn against a health app?
   e. What aspects of the MS condition should an app for health take into account to be effective?
   f. Let's say that you can have your perfect app to help your patients be more physically active, what does this app do?
3. Possible motivational aspects.
   a. How would you involved your patient's loved ones to help them being physically active?
   b. Is there any trick that you use to convince your patients to do things that they don't like doing?
   c. What strategies would you suggest to increase physical activity to a person with MS?

Concluding question

Of all the things we've discussed today, what would you say are the most important issues related to developing health technology to improve physical activity.
