# Supplementary material for: Exploring the Specific Needs of Persons with Multiple Sclerosis for mHealth Solutions for Physical Activity: Mixed-Methods Study
Source: JMIR Mhealth Uhealth. 2018 Feb 9;6(2):e37. doi: 10.2196/mhealth.8996 (PMC5889817; doi:10.2196/mhealth.8996)
Supplement: Multimedia Appendix 2 [file mhealth_v6i2e37_app2.pdf]

Amanda Palmer

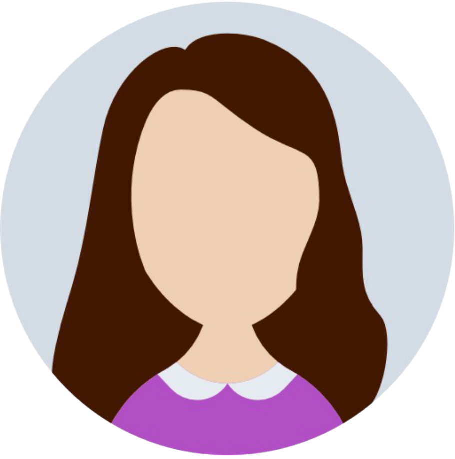

Bio

Amanda Palmer is 45 years old, she is divorced and not looking for a relationship. She works as a secretary in a law firm and doesn't like technology. She once went to an MS support group but didn't like the experience, ever since she is afraid of incontinence. She is not a sports fan and feels in sports in general are dumb. She feels that doing housework is enough physical activity. Friends are very important to her.

Amanda likes to plan her life but prefers to use a paper notepad. She is interested in more information about potential condition issues, such as pelvic exercises and different forms of therapy to manage incontinence.

"You have to want to do it, not have to do it."

Personal Information

Age: 47
Family: Divorced
Education: College degree
Occupation: Secretary

Medical Information

MS Type: Relapsing Remitting Multiple Sclerosis
Medications: Immunomodulators
Time since diagnosis: 15 years
EDSS Score: 3.0

Goals

- She wants to be strong, giving up is not an option

Frustrations

- People who are too competitive
- Doesn't like to talk about MS with others
- Things that are difficult to use

Personality

Introvert — Extrovert
Thinking — Feeling
Pessimist — Optimist
Cooperative — Competitive

Technology

Access
Interest
Trust
Fear

Physical Activity

Activity level
Disposition
Fear
Symptomatology

Device Ownership

Desktop PC | Mobile phone

ICT Use

Email | Social Media | Office Suite

John Peterson

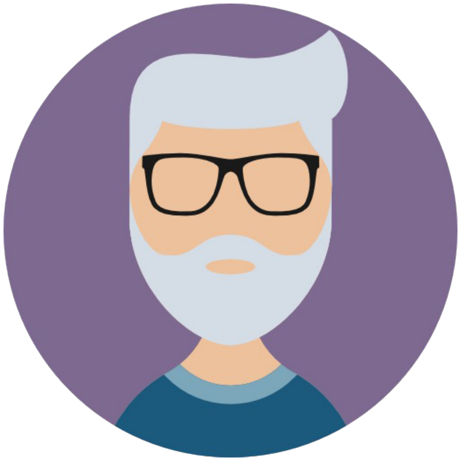

Bio

John Peterson is 38 years old and works in a small office. He lives with his wife Ivanna since they were 21. He loved being active but since he was diagnosed with MS, he is worried about doing too much and cause a relapse. He feels worn out all the time and tries to do most of his activities in the mornings so that he is done with them. He believes that one should train not only the body but also the mind. He has balance issues that hinder his coordination and his eyesight gets blurry sometimes. He enjoys the company of his family and has some friends with MS with whom he doesn't talk about his condition.

John doesn't like technology and he doesn't use apps; he says he can't see any real benefit for his condition since apps cannot change behaviours. He would give them a chance if his healthcare provider suggested it though. It's important that the app is accurate and he has 100% control over it. The app would need to have a lot of features to be valuable to him.

"I don't need an app to motivate me. Lazy people will keep being lazy."

Personal Information

Age: 38
Family: Married
Education: College degree
Occupation: Office Clerk

Medical Information

MS Type: Relapsing Remitting Multiple Sclerosis
Medications: Immunomodulators and antidepressants
Time since diagnosis: 12 years
EDSS Score: 4.0

Goals

- He wants to be able to manage his fatigue

Frustrations

- He can no longer do all the things he wants
- Technology doesn't make sense to him
- Exercising is hard work

Technology

Access

Interest

Trust

Fear

Personality

Introvert / Extrovert

Thinking / Feeling

Pessimist / Optimist

Cooperative / Competitive

Physical Activity

Activity level

Disposition

Fear

Symptomatology

Device Ownership

Laptop PC | Mobile phone | Video Camera

ICT Use

Email | Social Media | Office Suite

Laura Miller

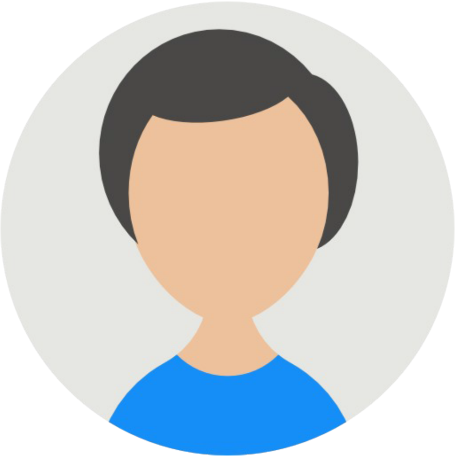

Bio

Laura Miller is 44 years old, she is married and has two kids. She works as a journalist for an online newspaper and is a very technological person. She loves to spend her free time with her loved ones. Family is very important to her, when she was diagnosed she started worrying whether she would become a burden to them. This is her prime motivation for staying active and managing her condition.

Laura takes 30 minutes walks every day, she wants to do more but is afraid it might cause a relapse. She keeps track of her steps with an app installed in her Smartwatch. Seeing how she progresses is very important to her. She would like it if there were more apps that are useful to her, especially ones that dealt with her condition. She feels most apps are too generic for her needs and wants them to give more personalized experiences. For example, she thinks it would be great if she could register her weaknesses in an app and the app could focus on training to strengthen these weaknesses.

"I have to stay active, I don't want to be a burden to my family."

Personal Information

Age: 44
Family: Married with 2 kids
Education: College degree
Occupation: Journalist

Medical Information

MS Type: Relapsing Remitting Multiple Sclerosis
Medications: Immunomodulators and analgesics
Time since diagnosis: 19 years
EDSS Score: 3.5

Goals

- She wants to live a normal life

Frustrations

- At the end of the day she feels very tired
- She is embarrassed about the way she walks
- Apps don't offer good enough experiences

Technology

Access
Interest
Trust
Fear

Personality

Introvert — Extrovert
Thinking — Feeling
Pessimist — Optimist
Cooperative — Competitive

Physical Activity

Activity level
Disposition
Fear
Symptomatology

Device Ownership

Desktop PC | Mobile phone | Laptop PC | Smartwatch

ICT Use

Email | Social Media | Office Suite | Photography | IM Messaging

Tim Smith

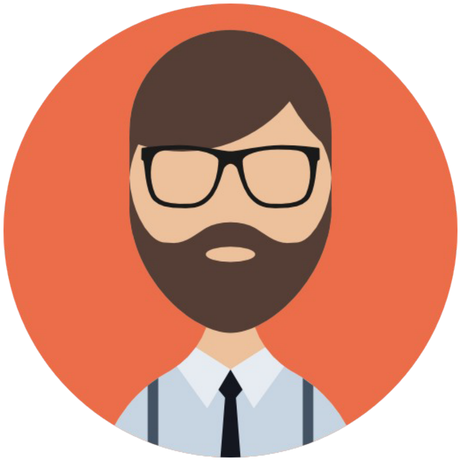

Bio

Tim Smith is 42 and teaches in an elementary school. He has been married for 17 years to Margaret and has a 15 year old son. He likes to go biking with him every Saturday. He feels fine in the morning but gets really frustrated about how tired he is in the afternoon. He has an app that helps record his steps and this has sparked his interest in technology. He now wants to try more and prefers apps that can suggest new exercises for him. However, he is worried about the privacy issues. He would like to know who can access his data before installing and using it.

Tim likes to keep a positive attitude, which is why when talking to other persons with MS he avoids talking about their condition. He likes being in online social groups. It's important for him to be with others and discuss his ideas.

"I love biking, there are some great apps to keep track of my activity."

Personal Information

Age: 42
Family: Married with 1 kid
Education: University degree
Occupation: Teacher

Medical Information

MS Type: Relapsing Remitting Multiple Sclerosis
Medications: Immunomodulators
Time since diagnosis: 5 years
EDSS Score: 4.5

Goals

- He wants to overcome all challenges

Frustrations

- Hates it when he feels tired
- People make fun of the way he walks
- App developers don't disclose all they should

Technology

Access

Interest

Trust

Fear

Personality

Introvert — Extrovert

Thinking — Feeling

Pessimist — Optimist

Cooperative — Competitive

Physical Activity

Activity level

Disposition

Fear

Symptomatology

Device Ownership

Laptop PC | Mobile phone

ICT Use

Email | Social Media | Office Suite | IM Messaging
